# Optimizing Liposuction in Lipedema Patients: A Novel Approach with Perioperative and Intraoperative Ultrasound

**DOI:** 10.1007/s00266-026-05889-x

**Published:** 2026-05-11

**Authors:** Joaquim Munoz, Soledad Fons, Mariachiara Fabbri

**Affiliations:** 1 Clinica Diagonal – Carrer de Sant Mateu, 24 - 08950 Barcelona, Spain; 2 Residency Program in Plastic Surgery, Università Cattolica del “Sacro Cuore” - Largo A. Gemelli 8, 00168 Rome, Italy

**Keywords:** Lipedema, Ultrasound, Liposuction, Fat

## Abstract

**Background:**

Lipedema is a chronic and progressive adipose tissue disorder that is often misdiagnosed and notoriously resistant to weight loss. Liposuction remains the most effective surgical treatment, but it requires precise technique to preserve the fragile lymphatic system. This study investigates the utility of pre-, intra- and postoperative ultrasound (US) to objectively assess fat reduction and the selective removal of pathological adipose tissue in patients undergoing liposuction for lipedema.

**Methods:**

A retrospective, single-center study of 24 female patients with lipedema who underwent liposuction of the lower extremities. Perioperative US was used to measure the thickness of the superficial subcutaneous fat (D1) and the deep fat layer (D2) at a standardized anatomical site. Intraoperative US was employed to verify that fat aspiration was performed in the correct superficial plane. A paired t-test was conducted to assess the statistical significance of the change in D1 thickness.

**Results:**

The mean patient age was 38 years, with a mean BMI of 25.3 kg/m^2^. The mean volume of liposuction aspirate was 4.5 L. Statistical analysis showed a significant reduction in mean D1 thickness from 9.9 mm preoperatively to 6.3 mm immediately postoperatively (*p* < 0,05). This reduction was sustained at the 3-month follow-up, with a mean D1 thickness of 5.8 mm.

**Conclusion:**

Our pilot study suggests that the perioperative use of ultrasound is a valuable tool for objectively documenting the selective fat reduction achieved with liposuction in lipedema patients. Intraoperative US not only enhances surgical precision, but also reduces the risk of complications by confirming correct cannula positioning in the superficial plane. This technique enhances surgical precision by allowing for the quantifiable removal of pathological superficial fat, confirming its potential to improve outcomes with a low complication rate.

**Level of Evidence IV:**

This journal requires that authors assign a level of evidence to each article. For a full description of these Evidence-Based Medicine ratings, please refer to the Table of Contents or the online Instructions to Authors  www.springer.com/00266.

**Supplementary Information:**

The online version contains supplementary material available at 10.1007/s00266-026-05889-x.

## Introduction

Lipedema is a chronic and progressive condition characterized by a bilateral and symmetrical accumulation of pathological adipose tissue, primarily in the legs and arms, while often sparing the feet and hands [1, 2]. It has been estimated that this disorder might affect nearly 1 out of 9 adult women worldwide [3]. Its onset is typically associated with hormonal changes, such as those occurring during puberty, pregnancy, or menopause, which has led to the hypothesis of a strong endocrine involvement in its pathogenesis [1]. Despite its high prevalence, lipedema remains often underdiagnosed or misdiagnosed as obesity or lymphedema.

The diagnosis of lipedema is primarily clinical, based on a comprehensive patient history and physical examination. Key diagnostic features include disproportionate fat distribution and tenderness upon palpation of the affected areas. A classic clinical sign is the preservation of fat-free areas around the ankles, often referred to as the “cuff sign,” which helps differentiate it from lymphedema [4, 5]. Lipedema is also characterized by easy bruising and a nodular or fibrous texture of the subcutaneous tissue [6]. The disease is categorized into several stages based on the severity and extent of fat accumulation and tissue changes [1, 7].

Current management of lipedema focuses on conservative and surgical approaches. Conservative treatments include an anti-inflammatory diet [8], exercise therapy [9], manual lymph drainage massages, and the use of compression garments [10, 11]. While these measures can help manage symptoms, they are often not sufficient to reduce the pathological adipose tissue, which is notoriously resistant to conventional weight loss methods. For this reason, surgical treatment, specifically liposuction, is considered the most effective long-term solution for reducing the pathological fat and improving patient quality of life [12, 13].

Liposuction for lipedema differs from cosmetic liposuction and requires specific technical expertise to preserve the lymphatic vessels, which are often fragile in these patients [5, 14]. A key challenge lies in precisely identifying and targeting the superficial fat compartment, while avoiding deeper layers where more critical structures are located. In particular, regions such as the anterior tibio-peroneal compartment and the medial ankle (posterior tibial bundle) require extra caution. This study aims to address this challenge by introducing a novel intraoperative ultrasound-guided liposuction technique [15]. We hypothesize that this technique allows for a more accurate identification of the correct surgical plane, enabling the selective removal of pathological adipose tissue from the superficial compartment. Furthermore, we will demonstrate its utility in providing objective pre- and post-operative evaluations, thereby enhancing surgical precision and improving outcomes for patients with lipedema.

## Materials and Methods

This retrospective, single-center study included 24 patients who underwent liposuction of the lower extremities for lipedema with preoperative and postoperative ultrasound (US) study of subcutaneous tissue thickness, between January 2025 and June 2025 in a private clinical setting. Exclusion criteria were lipedema type I, II, or IV, ASA > 3, and hemoglobin < 8 g/dL. All procedures were performed by the first author. The study was conducted in accordance with the Declaration of Helsinki, and all patients provided written informed consent.

*Preoperative management:* Patients were instructed to follow a strict anti-inflammatory diet beginning three months prior to surgery. Active smokers were advised to cease smoking at least one month before the procedure.

*Surgical procedure and ultrasound protocol:* All procedures were performed under regional anesthesia with sedation. A portable high-frequency ultrasound device (Clarius L15 HD3, Clarius Health®), set at 5–15 MHz with a maximum depth of 7 cm in musculoskeletal (MSK) mode, was employed from the very beginning of the operation. This allowed the surgeon to establish a precise baseline assessment and to continuously monitor the surgical field during fat aspiration.

Following anesthesia, a tumescent solution (physiologic saline with adrenaline) was infiltrated, with a waiting period of approximately 5 minutes before starting liposuction. The liposuction itself was conducted under real-time ultrasound guidance to ensure accurate selection of the surgical plane, particularly targeting the superficial compartment while avoiding deeper structures (Video 1). Special attention was given to critical anatomical regions such as the anterior tibio-peroneal compartment and the medial ankle, where the posterior tibial neurovascular bundle is located.

A power-assisted device was used with 3- and 4-mm Mercedes cannulas (straight and curved), while basket cannulas were deliberately avoided due to their traumatic effect on tissue. The procedure typically began with a 4-mm cannula introduced into a slightly deeper plane, progressively transitioning toward the superficial compartment. Final refinements were performed with a 3-mm cannula, restricted to the superficial fat layer only.

Before liposuction, an ultrasound baseline scan of the subcutaneous tissue was performed 4 cm cranial to the medial malleolus. Images were transmitted in real time to a mobile device through a dedicated application. Two measurements were systematically recorded:

- *D1:* Thickness (cm) of the superficial subcutaneous fat down to Scarpa’s fascia
- *D2:* Thickness (cm) of the deep fat layer, from Scarpa’s fascia to the underlying muscle fascia

At the end of the procedure, a postoperative ultrasound scan was repeated at the same anatomical site to reassess D1 and D2, confirming the selective reduction of the superficial fat layer.

*Postoperative protocol:* Patients were generally discharged on the first postoperative day with antibiotic and analgesic therapy, as well as prophylactic intramuscular heparin for 10-15 days in case of lipedema type V and for 20 days for lipedema type III. All patients were prescribed flat-knit class II medical compression garments, to be worn for a minimum of two and up to four months. From one month postoperatively, patients resumed regular physical activity. They were also instructed to maintain an anti-inflammatory diet and undergo a course of at least 10 sessions of manual lymphatic drainage, starting one week after surgery. In cases involving liposuction volumes exceeding 5 L, patients remained hospitalized for two nights, and a hemogram was performed 24 hours after surgery, prior to discharge. In two cases within the series, postoperative hemoglobin levels were borderline (8.5 g/dL), and both patients received transfusion of two units of packed red blood cells. (Note: patients with preoperative hemoglobin values below 8 g/dL were excluded from the study.)

*Follow-up:* Follow-up assessments were scheduled at 3 and 6 months postoperatively, with ultrasound performed at the same reference point to evaluate the long-term stability of fat removal. Measurements focused on D1 thickness at three time points: preoperative, immediate postoperative, and 3 months postoperative. In addition, ultrasound was used during follow-up visits to diagnose postoperative complications, like muscular hernias.

## Results

All 24 participants were female, with a mean age of 38 years (range 25–59) and an average body mass index (BMI) of 25,3 kg/m^2^ (range: 19,7–31,3). Lipedema classification (by Schrader) showed 22 cases of type III and 2 cases of type V. Two patients were smokers but discontinued one month before the surgery**.** All patients reported preoperative pain, which showed a significant reduction postoperatively. Functional improvement was observed in all patients, including better performance in daily activities and physical exercise. The mean operative time was 3 hours**.** Patients’ characteristics are showed in Table 1.

**Table 1:** Patients’ characteristics

Total n. of patients	24
Mean BMI	25,3 kg/m^2^(min: 19,7; max: 31,3)
Lipedema type	Type III: 22 patients
	Type V: 2 patients
Smoke	2

*Surgical outcomes:* The mean volume of liposuction aspirate was 4,5 L (range: 1,5–8L).

*Ultrasound (US) findings:* The minimum follow-up period was 3 months, the max 9 months. Preoperative mean total subcutaneous thickness was 16,7 mm (range: 9,5–26,3), with a mean D1 thickness of 9,9 mm (range: 5,4–16,2). Immediately postoperatively, mean total thickness decreased to 9,3 mm (range: 5,2–16,4), with D1 at 6,3 mm (range: 4,7–7,7). At 3 months, mean total thickness was 8,95 mm (range: 5,2–16,1), while D1 was 5,8 mm (range: 4,9–7,5).

Statistical analysis was conducted using a paired t-test to evaluate the change in D1 thickness. The results showed a statistically significant reduction in D1 thickness between preoperative and immediate postoperative measurements (*p* < 0,05). Surgical and ultrasound findings are displayed in Table 2.

**Table 2:** Surgical and US findings

Mean liposuction volume	4,5L(min: 1,5L; max: 8L)
Mean total subcutaneous thickness	**Preop:16,7 mm** (min: 9,5 mm; max: 26,3 mm)
	**Immediate postop:9,3 mm** (min: 5,2 mm; max: 16,4 mm)
	**3 months postop: 8,95 mm** (min: 5,2 mm; max: 16,1)
Mean D1 thickness	**Preop: 9,9 mm** (min: 5,4 mm; max: 16,4 mm)
	**Immediate postop: 6,3 mm** (min: 4,7 mm; max: 7,7 mm)
	**3 months postop: 5,8 mm** (min: 4,9 mm; max: 7,5)

Bold highlights the measurements taken preoperative, immediate post operative and at 3 months postoperative

**Fig. 1:**
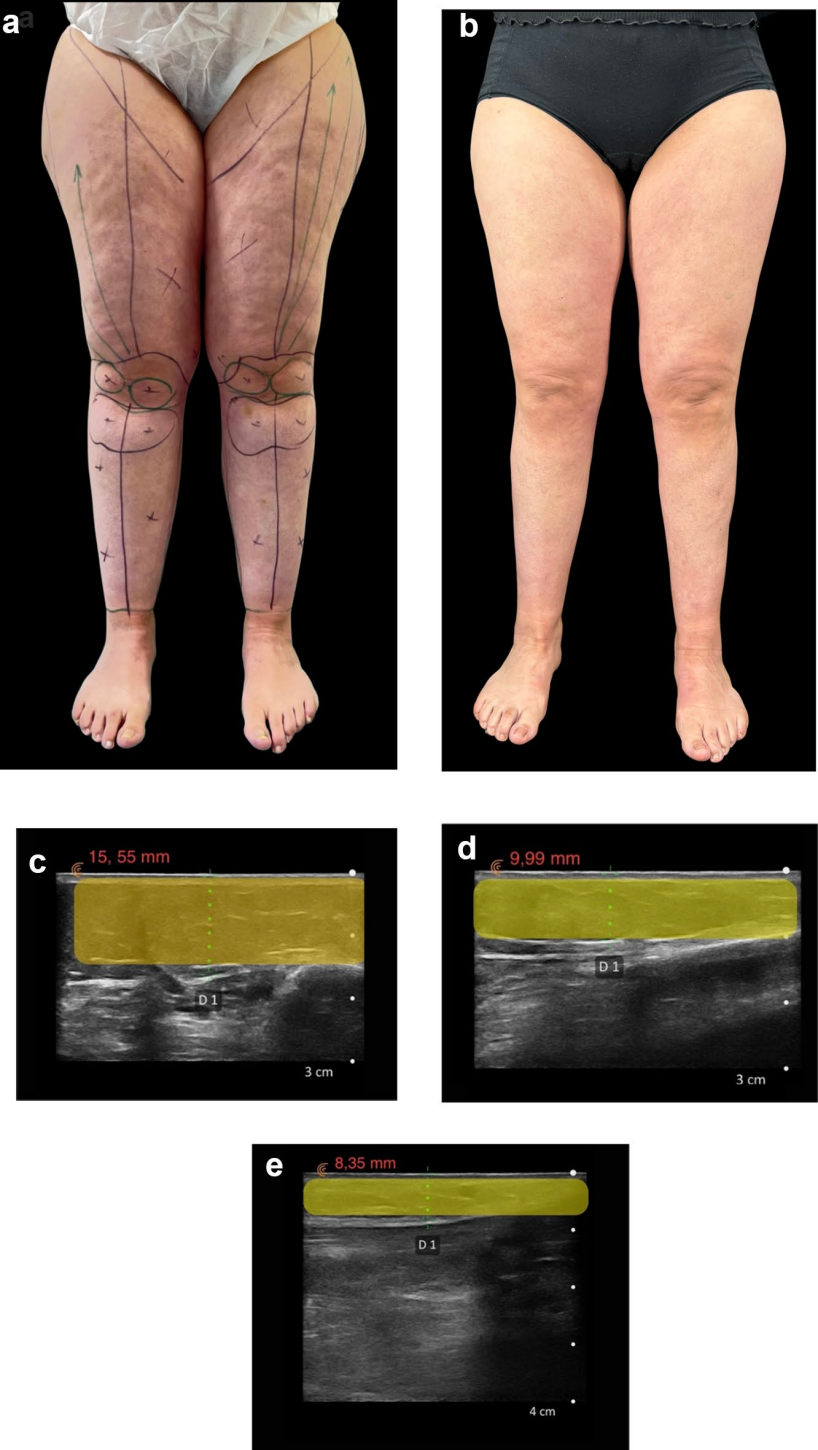
41 y.o. female patient with type III lipedema; **a** preoperative frontal view; **b** 3 months post operative frontal view; **c** Preoperative D1 thickness; **d** Immediate postoperative D1 thickness; **e** 3 months post operative D1 thickness

**Fig. 2:**
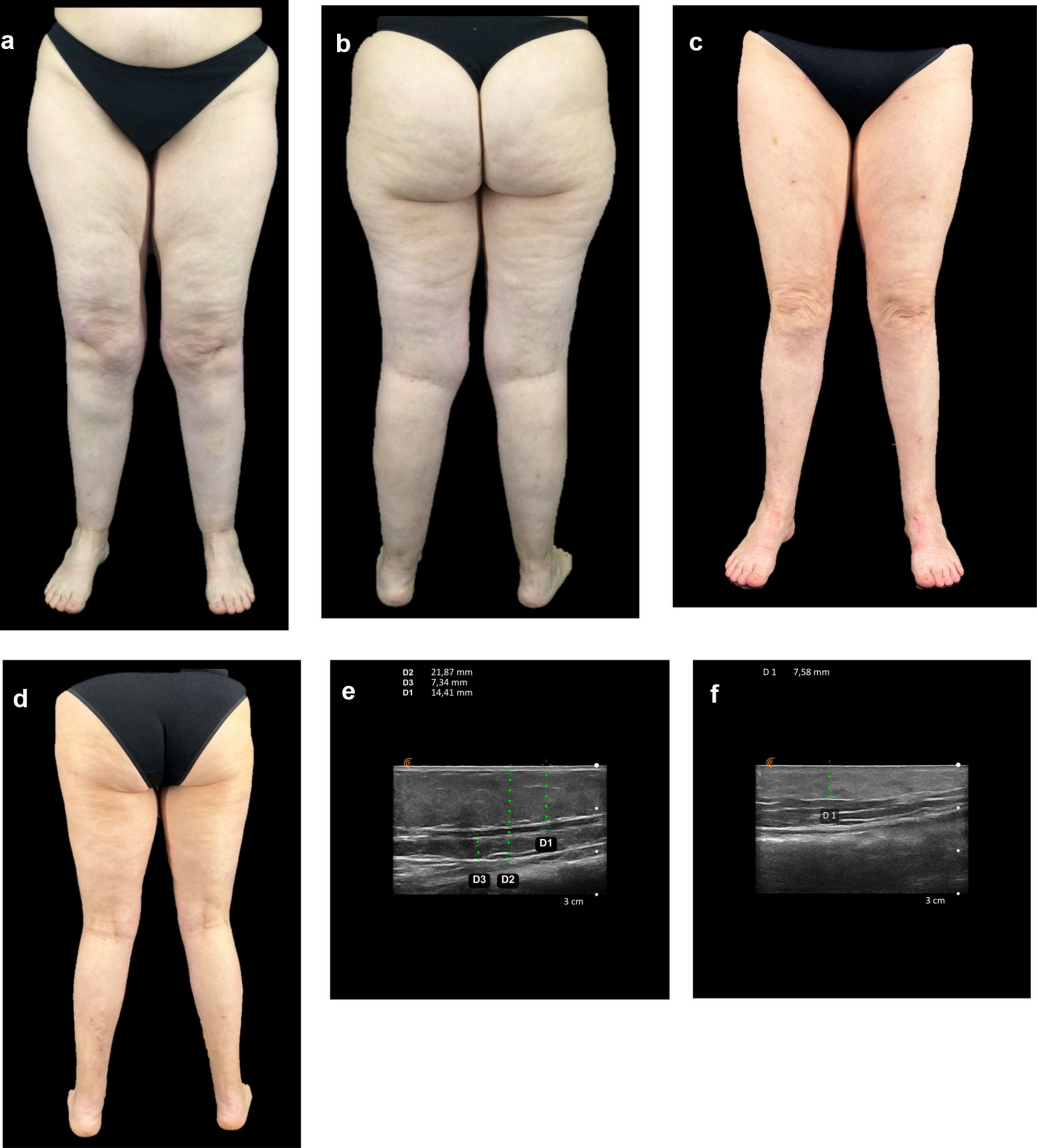
46 y.o. female patient with type III lipedema; **a** preoperative frontal view; **b** Preoperative back view; **c** 3 months post-operative frontal view; **d** 3 months post-operative back view **e** Preoperative D1 and D2 thickness; **f** 3 months post operative D1 thickness

**Fig. 3:**
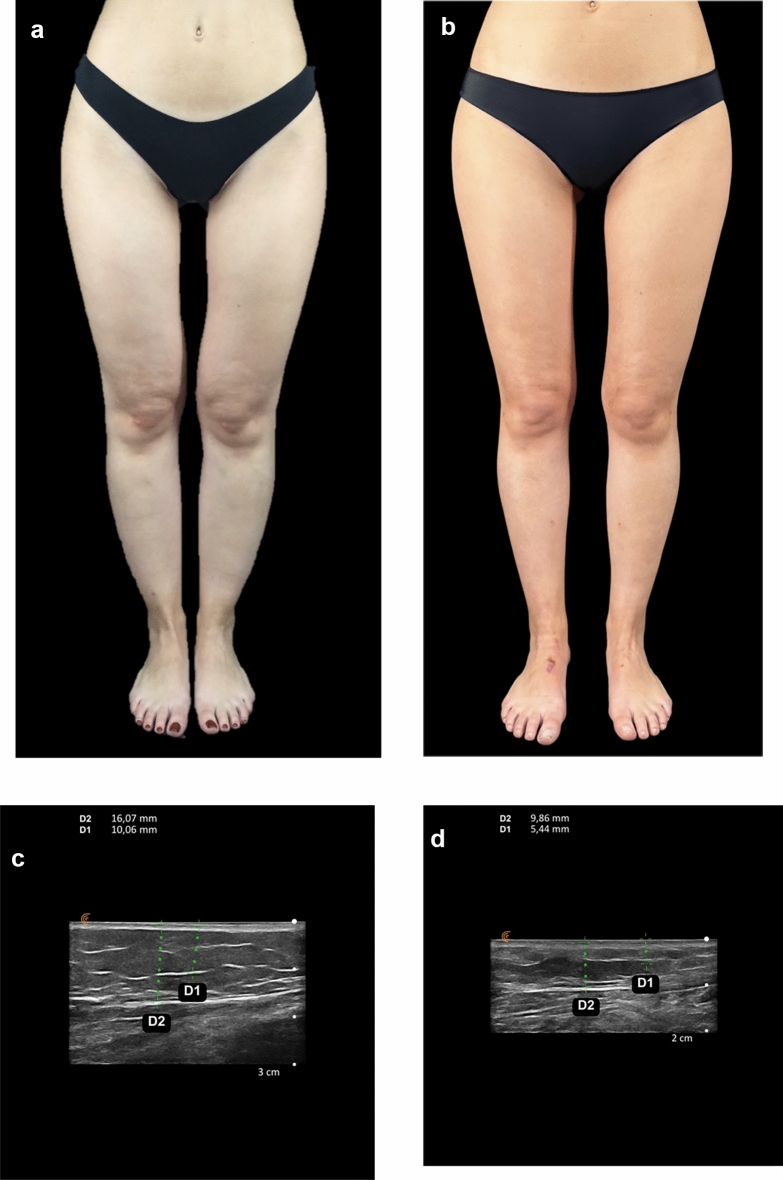
29 y.o. female patient with type V lipedema; **a** preoperative frontal view; **b** 6 months post-operative frontal view; **c** Preoperative D1 and D2 thickness; **d** 6 months post-operative D1 and D2 thickness

Please refer to Figs. 1, 2 and 3 for preoperative and postoperative pictures.

*Complications:* Three patients experienced complications: one pulmonary thromboembolism despite heparin prophylaxis (managed conservatively), one transfusion reaction treated with corticosteroids, and one muscular hernia at the tibial level, diagnosed by ultrasound and surgically repaired under local anesthesia.

## Discussion

Lipedema is a chronic and progressive condition that remains a diagnostic and therapeutic challenge. Our study demonstrates the feasibility and potential benefits of integrating perioperative ultrasound guidance into liposuction procedures for lipedema patients. The use of ultrasound in our series of patients allowed for precise identification of the superficial fat compartment, confirming the selective removal of pathological adipose tissue, as evidenced by the significant reduction in D1 thickness, while preserving deeper structures and lymphatics.

First of all, our results suggest that lipedema is a condition independent of obesity, as most of our patients did not fall within the obese weight range (average BMI of 25.3 kg/m^2^ with a range from 19.7 to 31.3). This finding is consistent with previosu literature suggesting that lipedema is characterized by pathological adipose deposition that is largely independent of generalized obesity.

The effectiveness of liposuction in improving symptoms and quality of life in lipedema patients has already been demonstrated in several studies. However, most published series focus primarily on surgical outcomes and symptom improvement without incorporating real-time imaging guidance during the procedure. Our study adds to the current literature by introducing the use of intraoperative ultrasound as a tool to identify the pathological fat plane and guide selective aspiration throughout the procedure. Real-time ultrasound guidance provides several potential advantages. First, it allows accurate identification of the adipose layer with the greatest pathological accumulation, which in lipedema is typically located in the superficial compartment. This facilitates targeted fat removal, improving surgical efficiency and potentially optimizing the volume of adipose tissue removed during the operative time. Second, ultrasound guidance may help avoid inadvertent injury to deeper structures such as fascia, muscles, and neurovascular bundles. Damage to these structures may lead to complications such as muscular hernias, which can negatively affect aesthetic outcomes and may require additional surgical repair. By maintaining the cannula within the superficial plane, ultrasound guidance may contribute to safer and more controlled liposuction. Previous studies have evaluated different liposuction techniques for lipedema management. For instance, a large cohort study by Barbara Hersant and colleagues [16] reported favorable outcomes using ultrasound-assisted liposuction with VASER technology in a large series of patients. Similarly, the study by Nicolás Pereira and colleagues [17] investigated selective combined liposuction and reported significant improvements in symptoms and aesthetic perception. However, these approaches did not incorporate real-time ultrasound imaging to identify the surgical plane during the procedure. An additional interesting observation in our series is that the entire lower limb could be treated in a single operative session. In contrast, previous studies have reported staged procedures or the need for additional interventions in a proportion of patients. For example, Pereira et al. [18] reported that approximately 25% of patients required a second surgical procedure. In contrast to these previously published studies, the only technology used in our series was power-assisted liposuction (PAL), without the use of energy-based devices such as VASER. Despite the absence of additional energy-assisted technologies, our series demonstrated a postoperative seroma rate of 0%, suggesting that precise ultrasound-guided identification of the superficial adipose plane combined with controlled mechanical aspiration may contribute to minimizing postoperative fluid collections. Please refer to Table 3 for more information regarding the different liposuction techniques.

**Table 3:** Comparison between previously published liposuction approaches for lipedema and the ultrasound-guided technique proposed in the present study

Study	Study design	Technique	Use of us	Surgical strategy	Main outcomes
Hersant et al., 2025	Single-center cohort, 191 patients	VASER-assisted liposuction	Ultrasound energy device but no real-time imaging guidance	Often staged procedures depending on limb involvement	Significant improvement in symptoms and limb contour
Pereira et al.	Clinical series	Selective Combined Liposuction	No intraoperative ultrasound guidance	In some cases, multiple procesdures required (≈ 25%)	Improvement in symptons and aesthetic self perception
Present study	Retrospective study, 24 patients	Ultrasound-guided power assisted liposuction	Real-time intraoperative ultrasound sed to identify superficial fat plane	Whole limb treated in a single procedure	Objective reduction of superficial fat layer (D1) improved surgical precision, low complication rate

In our cohort, the use of ultrasound guidance may have facilitated a more targeted and efficient fat removal, allowing treatment of the whole limb in a single stage. Beyond intraoperative guidance, ultrasound also demonstrated additional clinical value during postoperative follow-up. In our series, it enabled the early diagnosis of complications such as muscular hernias during routine outpatient assessment, reinforcing its role as both a surgical and diagnostic tool in the management of lipedema.

This dual diagnostic and intraoperative role strengthens the position of ultrasound as an indispensable tool in lipedema surgery. The data confirm a statistically significant reduction in the thickness of the superficial layer (D1) from preoperative (mean 9.9 mm) to immediate postoperative (mean 6.3 mm), with a *P* value < 0.05. The low complication rate observed in our series suggests that this technique is not only effective but also safe.

This study has several limitations. First, its retrospective design and relatively small sample size limit the generalizability of the findings. Second, the absence of a control group undergoing conventional liposuction without ultrasound guidance prevents direct comparison between techniques. Third, ultrasound measurements were performed at a standardized anatomical point, which may not fully represent fat distribution across the entire treated limb. Finally, the follow-up period remains relatively short, and longer-term prospective studies with larger cohorts are necessary to further validate these findings and better assess long-term outcomes.

## Conclusion

In conclusion, our pilot study suggests that the use of perioperative and intraoperative ultrasound guidance is a valuable tool for optimizing liposuction outcomes in lipedema patients. This technique enhances surgical precision by allowing for the selective removal of pathological fat from the superficial compartment, with a statistically significant effect that we were able to quantify with objective ultrasound measurements. Intraoperative US also contributes to improved safety by directing cannula placement in high-risk anatomical areas and by reducing the incidence of complications such as muscular hernias, while postoperative ultrasound remains a reliable diagnostic aid for their early detection. Our short-term results support the use of ultrasound as a means to improve surgical safety and efficacy, potentially leading to better patient outcomes and reduced complication rates.

## Supplementary Information

Below is the link to the electronic supplementary material.

Video 1: Intraoperative US showing the passage of the cannula into the superficial fat compartment
